# *WFS1* autosomal dominant variants linked with hearing loss: update on structural analysis and cochlear implant outcome

**DOI:** 10.1186/s12920-023-01506-x

**Published:** 2023-04-11

**Authors:** Hui Dong Lim, So Min Lee, Ye Jin Yun, Dae Hee Lee, Jun Ho Lee, Seung-Ha Oh, Sang-Yeon Lee

**Affiliations:** 1 Department of Otorhinolaryngology-Head and Neck Surgery, Seoul National University Hospital, Seoul, Republic of Korea; 2 Department of Genomic Medicine, Seoul National University Hospital, Seoul, Republic of Korea; 3 CTCELLS, Inc, 21, Yuseong-daero, 1205beon-gil, Yuseong-gu, Daejeon, Republic of Korea

**Keywords:** *WFS1*, DFNA6/14/38, Wolfram-like syndrome, Structure analysis, Cochlear implantation

## Abstract

**Background:**

Wolfram syndrome type 1 gene (*WFS1*), which encodes a transmembrane structural protein (wolframin), is essential for several biological processes, including proper inner ear function. Unlike the recessively inherited Wolfram syndrome, *WFS1* heterozygous variants cause DFNA6/14/38 and wolfram-like syndrome, characterized by autosomal dominant nonsyndromic hearing loss, optic atrophy, and diabetes mellitus. Here, we identified two *WFS1* heterozygous variants in three DFNA6/14/38 families using exome sequencing. We reveal the pathogenicity of the *WFS1* variants based on three-dimensional (3D) modeling and structural analysis. Furthermore, we present cochlear implantation (CI) outcomes in *WFS1*-associated DFNA6/14/38 and suggest a genotype-phenotype correlation based on our results and a systematic review.

**Methods:**

We performed molecular genetic test and evaluated clinical phenotypes of three *WFS1*-associated DFNA6/14/38 families. A putative WFS1–NCS1 interaction model was generated, and the impacts of *WFS1* variants on stability were predicted by comparing intramolecular interactions. A total of 62 *WFS1* variants associated with DFNA6/14/38 were included in a systematic review.

**Results:**

One variant is a known mutational hotspot variant in the endoplasmic reticulum (ER)-luminal domain WFS1(NM_006005.3) (c.2051 C > T:p.Ala684Val), and the other is a novel frameshift variant in transmembrane domain 6 (c.1544_1545insA:p.Phe515LeufsTer28). The two variants were pathogenic, based on the ACMG/AMP guidelines. Three-dimensional modeling and structural analysis show that non-polar, hydrophobic substitution of Ala684 (p.Ala684Val) destabilizes the alpha helix and contributes to the loss of WFS1-NCS1 interaction. Also, the p.Phe515LeufsTer28 variant truncates transmembrane domain 7–9 and the ER-luminal domain, possibly impairing membrane localization and C-terminal signal transduction. The systematic review demonstrates favorable outcomes of CI. Remarkably, p.Ala684Val in *WFS1* is associated with early-onset severe-to-profound deafness, revealing a strong candidate variant for CI.

**Conclusions:**

We expanded the genotypic spectrum of *WFS1* heterozygous variants underlying DFNA6/14/38 and revealed the pathogenicity of mutant WFS1, providing a theoretical basis for WFS1-NCS1 interactions. We presented a range of phenotypic traits for *WFS1* heterozygous variants and demonstrated favorable functional CI outcomes, proposing p.Ala684Val a strong potential marker for CI candidates.

**Supplementary Information:**

The online version contains supplementary material available at 10.1186/s12920-023-01506-x.

## Introduction

Congenital hearing loss is the most common inherited sensory defect, with a prevalence of 1.2 to 1.7 newborns per 1,000 live births [1]. Developments in genetics have accelerated our understanding of the pathophysiology of congenital sensorineural hearing loss (SNHL), of which over 50% has a genetic etiology [2]. More than 200 genes and > 150 different loci have been identified as contributors to hereditary hearing loss (https://hereditaryhearingloss.org/) [3]. The genetic etiology aids our understanding of some types of genetic hearing loss in terms of clinical progress and application of optimized audiologic rehabilitation [4–12]. Moreover, functional classifications of genetic hearing loss, based on tonotopic expression patterns in the inner ear, as well as molecular insights from genetically engineered models, suggest promising approaches for targeted drug and gene therapy [13]. Interestingly, a few deafness-related genes with distinct phenotypes exist depending on the genotype and inheritance pattern. Thus, a thorough analysis of the clinical profiles and genotypes of these rare genes related to deafness is essential. *WFS1* is a good example of this type of gene.

Wolfram syndrome type 1 gene (*WFS1*), located on chromosome 4p16.1, encodes wolframin, which is a transmembrane protein consisting of 890 amino acids [14]. Although there have been controversial reports about the N-terminal and transmembrane (TM) localization of wolframin, the literature is consistent with respect to the sequence information of the cytoplasmic domain, TM domains 6–9, and the endoplasmic reticulum (ER)-luminal domain [15–17]. Wolframin is predominantly expressed in the ER and plays a vital role in membrane trafficking, post-translational modification, and maintaining the calcium homeostasis of endoplasmic reticulum [18, 19]. Although the pathophysiological mechanism remains elusive, defects of wolframin caused by pathogenic *WFS1* variants elicit altered post-translational modifications, such as unfolded proteins and ER stress, resulting in apoptosis [19]. Various phenotypes are attributable to its ubiquitous expression [20]. Wolframin is also expressed throughout the inner ear, including in the scala media, spiral ganglion, and hair cells [21]. In particular, it is localized in the canalicular reticulum, a specialized form of ER, suggesting a role in inner ear ion homeostasis [21]. Additionally, wolframin immunoreactivity has been detected in the basal cells of the stria vascularis in primates, in contrast to previous findings in mice [22]. These inter-species differences in wolframin expression may contribute to distinct phenotypes observed between species.

*WFS1* heterozygous variants have been reported to cause DFNA6/14/38 and wolfram-like syndrome, which is characterized by autosomal dominant nonsyndromic hearing loss (ADNSHL), optic atrophy and diabetes mellitus [23]. Neurologic dysfunctions such as vestibular impairments are not observed [24]. In contrast, recessively inherited variants in *WFS1* are responsible for Wolfram syndrome type 1, also known as DIDMOAD syndrome (diabetes insipidus, diabetes mellitus, optic atrophy, and deafness) [25]. More than 50 different heterozygous variants in *WFS1* have been shown to cause DFNA6/14/38, and most of the variants are present in the ER-luminal domain [26]. The phenotypic spectrum of DFNA6/14/38 and wolfram-like syndrome is highly heterogenous [27]. Moreover, the phenotype of DFNA6/14/38 varies among affected subjects in terms of its onset, severity and audiometric configuration [28], hampering a genotype-phenotype correlation. Additional reports and systematic reviews may enhance our understanding of *WFS1* heterozygous variants underlying DFNA6/14/38.

In this study, we report two *WFS1* heterozygous variants in three DFNA6/14/38 families via exome sequencing. One is a known mutational hotspot variant in the ER-luminal domain (c.2051 C > T:p.Ala684Val), and the other is a novel frameshift variant in transmembrane domain 6 (c.1544_1545insA:p.Phe515LeufsTer28). We reveal the pathogenicity of the *WFS1* variants based on three-dimensional (3D) modeling and structural analysis. Furthermore, we present cochlear implantation (CI) outcomes in *WFS1*-associated DFNA6/14/38 and suggest a genotype-phenotype correlation based on our results and a systematic review.

## Materials and methods

### Participants

This study was approved by the Institutional Review Board of Seoul National University Hospital (IRB-H-0905-041-281). Written informed consent was obtained from all participants or the legal guardians of the pediatric participants. We conducted a retrospective review using the in-house database of genetic hearing loss from a single tertiary hospital. Among 364 probands that went through molecular genetic testing regardless of audiologic phenotype and mode of inheritance, probands for which a causative *WFS1* heterozygous variant was identified were included. Ultimately, three *WFS1*-associated DFNA6/14/38 families, segregating as a dominant trait, were identified. We present the clinical phenotypes, genotypes, radiological imaging, and audiological rehabilitation of affected probands.

### Audiological evaluation

Hearing thresholds were measured using pure-tone audiometry (PTA) for six octave frequencies (0.25, 0.5, 1, 2, 4, and 8 kHz). In cases where PTA was not available for young children, the auditory steady-state response (ASSR) and bone-conduction/click auditory brainstem response (ABR) were utilized to determine the hearing thresholds. The mean hearing threshold was calculated as the average of the thresholds at 0.5, 1, 2, and 4 kHz measured by PTA and ASSR, and the degree of the hearing loss was divided into four categories. The mean hearing threshold was determined as the average of the thresholds at 0.5, 1, 2, and 4 kHz, and the degree of the hearing loss was classified into four categories based on the ASHA standard [29, 30]: mild (20–40 dB), moderate (41–70 dB), severe (71–90 dB), and profound (> 90 dB). Furthermore, audiological configuration was classified as high-frequency (4 and 8 kHz), mid-frequency (1 and 2 kHz), low-frequency (0.25 and 0.5 kHz), or flat. The audiologic performance of each cochlear implantee was evaluated by comparing the Categories of Auditory Perception (CAP) and/or speech perception tests, as appropriate based on age, preoperatively and postoperatively. Auditory perception performance was assessed according to eight categories, with CAP scores, using a hierarchical scale from 0 to 7 for children’s developing auditory abilities [31]. In addition, the Infant-Toddler Meaningful Auditory Integration Scale (IT-MAIS) and Sequenced Language Scale for Infants (SELSI) were examined. We also obtained pre- and postoperative comparative data of speech perception tests through word (monosyllabic [32] words and bisyllabic [spondee] words) and sentence-recognition tasks (K-CID; Korean version of the Central Institute of Deafness) at 70 dB SPL in an audio-only condition, particularly in adult cochlear implantees [12].

### Molecular genetic testing

Genomic DNA was extracted from peripheral blood using a standard procedure and subjected to initial screening with real-time PCR mutational hotspot screening kits targeting 22 variants of 10 hearing loss genes (*GJB2, SLC26A4, CDH23, TMPRSS3, MT-RNR1, OTOF, MPZL2, TMC1, COCH, and ATP1A3*).[4, 11] If these data were inconclusive, whole-exome sequencing was conducted to define the underlying molecular genetic etiology. Reads were aligned using the University of California Santa Cruz hg19 reference genome browser (https://genome.ucsc.edu/) running Lasergene ver. 14 software (DNASTAR, Madison, WI, USA). As described previously,[4–10] stepwise filtering strategies were adopted to retrieve genetic variants. Candidate variants were validated employing Sanger sequencing, and segregation studies were performed using parental DNA samples. All variants identified were classified in accordance with the ACMG/AMP guidelines for hearing loss [33, 34].

### Structural modeling

AlphaFold Protein Structure Database generated the model structure of WFS1 [35, 36]. To investigate the structural changes caused by truncated variants, the model with the highest structural accuracy was extracted using the Colabfold engine (https://github.com/sokrypton/ColabFold) [37]. A putative WFS1–NCS1(4GUK) interaction model was generated by PyDock algorithm for the rigid-body docking prediction of protein–protein complexes [38]. The mutagenesis of *WFS1* was determined using the Dynamut server (http://biosig.unimelb.edu.au/dynamut/) and PyMOL software (v.2.4.1). The impacts of *WFS1* variants on stability were predicted by comparing intramolecular interactions, such as cation-π interaction. The PyMOL program (v. 2.4.1; PyMOL Molecular Graphics System v. 2.0, Schrödinger Inc., New York, NY, USA) was used to create the figures.

## Results

### Clinical profiles

**Fig. 1:**
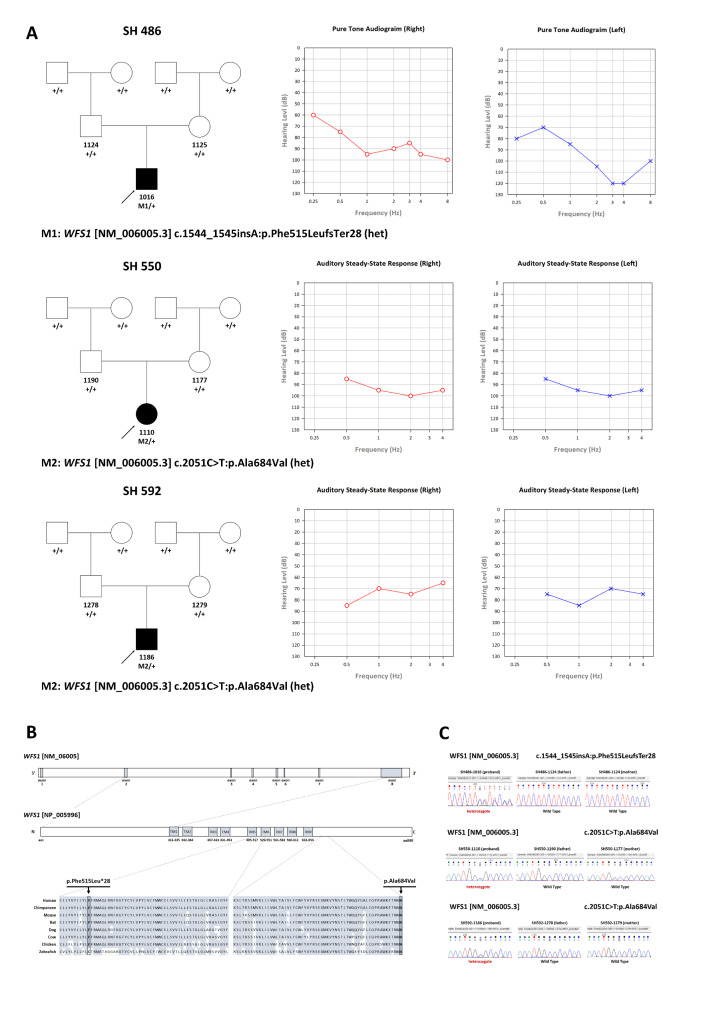
A schematic overview of the WFS1 protein, pedigrees of the three families, the audio-logical phenotypes of affected probands, and Sanger sequencing traces of the *WFS1* variants. **(A)** Pedigrees of three families with *WFS1* heterozygous variants and associated audiograms. **(B)** Physical map of *WFS1*, which contains nine transmembrane domains and an ER-luminal domain. The domains are represented as in the Universal Protein Resource (UniProt) database. The novel frameshift variant in SH486 (c.1544_1545insA:p.Phe515LeufsTer28) and the missense variant in SH550 andSH592 (c.2051 C > T:p.Ala684Val) reside in TM domain 6 and the ER-luminal domain, respectively. Conservation of the corresponding residues between species is depicted. **(C)** Sanger chromatogram of the respective *WFS1* heterozygous variants. All probands were confirmed as *de novo* occurrences

The demographics and clinical profiles of the three probands with *WFS1* variants are described in Table 1. The audiograms of each proband are depicted in Fig. 1A. In the SH486 family, the proband (SH486-1016: p.Phe515LeufsTer28) was associated with hearing impairment with prelingual onset (age of 4 years). The hearing loss deteriorated, revealing symmetric profound bilateral hearing loss with a high-frequency dominant configuration. Currently, the patient (SH486-1016) has undergone unilateral CI using a slim straight electrode (CI622) via the round-window approach. Compared to preoperatively, the speech perception scores were improved by 30 (PB word), 30 (Spondee word), and 30 (K-CID sentence) at postoperative 3 months (Additional file 1: Table S1). In the SH550 family, the proband (SH550-1110: p.Ala684Val) exhibited bilateral SNHL with congenital onset, with severe-to-profound severity across all frequencies. Both ears failed the newborn hearing screening test using automated auditory brainstem response. The proband (SH550-1110) underwent bilateral CI using a slim modiolar electrode (CI632) via a round-window approach at the age of 2 years, due to delayed language development and speech perception. The CAP score improved by 2 (from 1 to 3), and IT-MAIS improved by 36 points (from 2 to 38) at postoperative 3 months. Furthermore, significant improvements in receptive and expressive language ability were noted (Additional file 1: Table S1). In the SH592 family, the proband (SH592-1186: p.Ala684Val) was also associated with hearing impairment with congenital onset. Upon auditory brainstem response threshold and auditory steady state response tests, symmetric severe-to-profound SNHL across all frequencies was documented in both ears; thus, bilateral CI was scheduled. Meanwhile, based on the latest evaluations, none of the three probands exhibited symptoms of wolfram-like syndrome such as vision loss, optic atrophy, and diabetes mellitus, except for hearing impairment. Additionally, abnormalities of neither the inner ear nor brain were observed in temporal bone computed tomography (CT) and magnetic resonance imaging of the internal acoustic canal (IAC MRI).

**Table 1:** Demographics of the probands in the present study

Family	Sex	Age	Genotypes	Hearing Loss				Other phenotypes			
				Onset (yr)	Severity*	Configuration		Optic atrophy	Diabetes mellitus	Neurologic Examination	Age of Cochlear Implant
SH 486	M	56	c.1544_1545insA	4	profound	HF		normal	normal	normal	56
SH 550	F	34mo	c.2051 C > T	1	severe	flat		normal	normal	normal	29mo
SH 592	M	13mo	c.2051 C > T	1	severe	flat		normal	normal	normal	Scheduled

LF: low-frequency sensorineural hearing loss, MF: middle-frequency sensorineural hearing loss, HF: high-frequency sensorineural hearing loss, N/A: not available;

* Severity: mild(20-40dB), moderate(41-70dB), severe(71-90dB), profound(> 90dB).

### Genotypes

All probands’ genomic DNA went through comprehensive molecular genetic testing. Two heterozygous variants of *WFS1*-associated ADNSHL were identified: c.1544_1545insA:p.Phe515LeufsTer28 and c.2051 C > T:p.Ala684Val. The novel frameshift variant (c.1544_1545insA:p.Phe515LeufsTer28) in transmembrane domain 6 was truncated at premature stop codon at 543, which is predicted to undergo nonsense-mediated mRNA decay (NMD). The previously reported missense variant (c.2051 C > T:p.Ala684Val) was located in the ER-luminal domain (Fig. 1B). The two variants were extremely rare in several genome databases, such as the Korean Reference Genome Database (1,722 individuals) (https://coda.nih.go.kr/coda/KRGDB) and the Global Minor Allele Frequency database, including the Exome Aggregation Consortium (http://exac.broadinstitute.org/) and genome aggregation database (http://gnomad.broadinstitute.org/). Furthermore, the amino acid residues of Phe515 and Ala684 were highly conserved among *WFS1* orthologs in a diverse range of species, with Genomic Evolutionary Rate Profiling (GERP++) scores of 4.38 and 5.49, respectively. Specifically, p.Ala684Val had a higher *in silico* impact based on Combined Annotation Dependent Depletion (CADD) (https://cadd.gs.washington.edu/) and Rare Exome Variant Ensemble Learner (REVEL) (https://sites.google.com/site/revelgenomics/) algorithms, with scores of 28.8 and 0.891, respectively. Functional research has established the pathogenicity of the p.Ala684Val variant [19] and alternative variant (p.Ala684Thr) with corresponding residues [39, 40]. Co-segregation analysis confirmed that the two variants segregated as *de novo* trait in three unrelated families (Fig. 1C). Based on the ACMG/AMP rules on hearing loss, both variants identified herein are pathogenic (Table 2).

**Table 2:** *WFS1* variants in the present study and its pathogenicity prediction analysis

Family	Genomic Position	HGVS				In Silico Prediction				MAF					ACMG/AMP 2018 Guideline	
		Coding DNA Change	Protein Change	Domain	Zygosity	CADD	REVEL	GERP		KRGDB (1722 individuals)	ExAC	gnomAD			Criteria	Classification
SH 486	Chr4: 6,303,067	c.1544_1545insA	p.Phe515LeufsTer28	TM6	Het	N/A	N/A	4.38		Absent	Absent	Absent			PVS1 PS2_Sup. PM2 PP4	Pathogenic
SH 550 SH 592	Chr4: 6,303,067	c.2051 C > T	p.Ala684Val	ER lumen	Het	28.8	0.891	5.49		Absent	Absent	0.000007			PS1 PS2_Sup. PM2 PP3 PP4	Pathogenic

Refseq transcript accession number NM_006005.3; Refseq protein accession number NP_005996.2.

HGVS: Human Genome Variation Society (https://www.hgvs.org/); Sequence Variant Nomenclature (http://varnomen.hgvs.org/); CADD: Combined Annotation Dependent Depletion (https://cadd.gs.washington.edu/); REVEL: Rare Exome Variant Ensemble Learner (https://sites.google.com/site/revelgenomics/); KRGDB: Korean Reference Genome Database (http://coda.nih.go.kr/coda/KRGDB/index.jsp); ExAC: Exome Aggregation Consortium databases; gnomAD: The Genome Aggregation Database (https://gnomad.broadinstitute.org/); ACMG/AMP 2018 guideline (http://wintervar.wglab.org/).

TM indicates transmembrane; Het, heterozygote; MAF, minor allele frequency; N/A, not available.

### 3D modeling and structural analysis

**Fig. 2:**
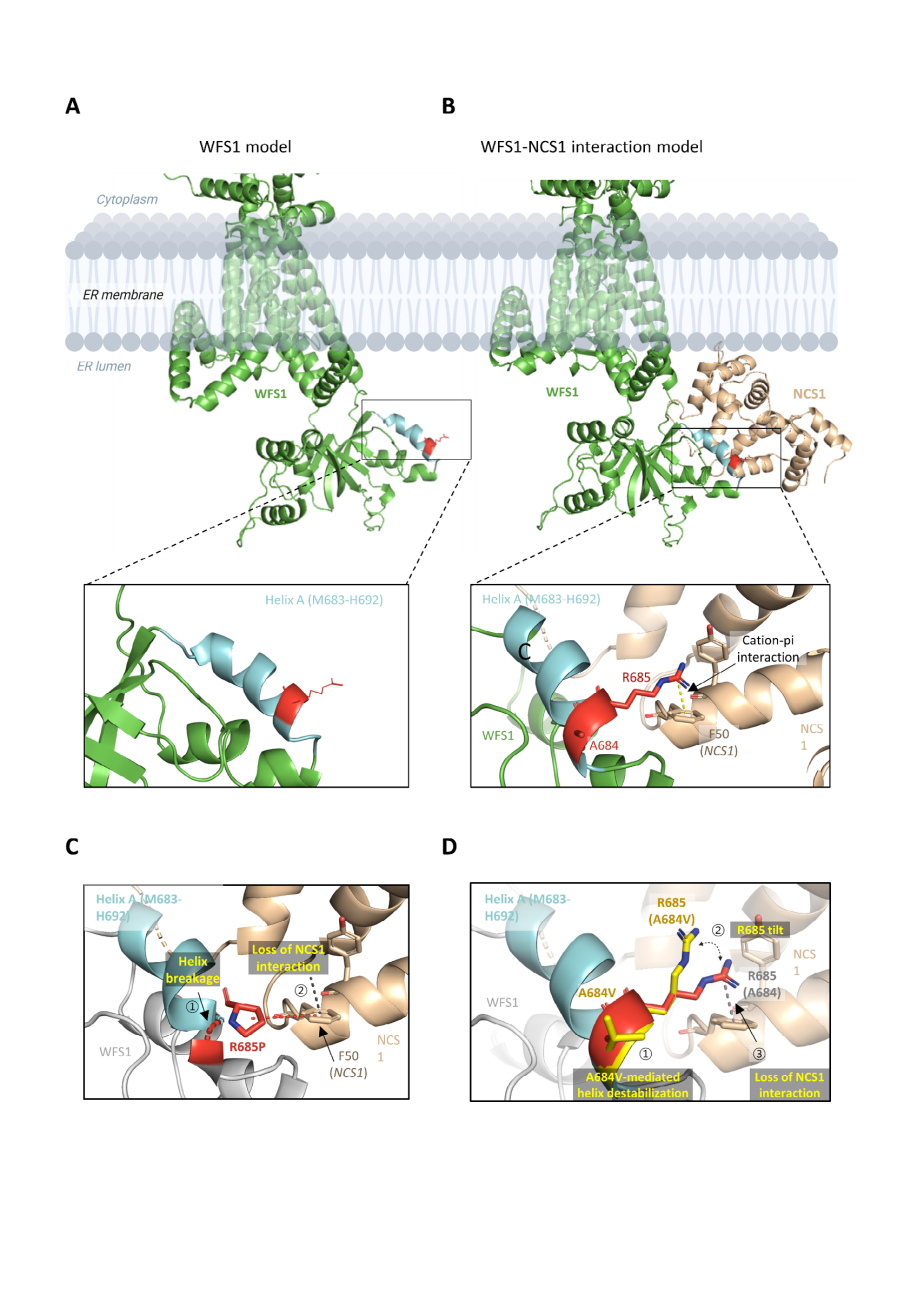
3D modeling and structural analysis of WFS1 p.Ala684Val. **(A)** WFS1 3D model generated from Alphafold (green). Ala684/Arg685 are located at the alpha-helix (Cyan, Met683-His692) of the ER-luminal domain. **(B)** Putative WFS1(Alphafold model) – NCS1(4GUK) interaction model generated by PyDock software. Arg685 extrudes from alpha helix (helix A) and directly interacts with NCS1 Phe50 via cation-π interaction (black dashes). **(C)** Loss of WFS1-NCS1 interaction in p.Arg685Pro. The p.Arg685Pro mutant loses its own cation- π interaction, which is required for WFS1-NCS1 interaction. Moreover, proline substitution breaks helix A [1], contributing to the loss of NCS1 interaction [2]. **(D)** Non-polar, hydrophobic substitution of A684 induces helix destabilization and distorts helix A [1]. The side chain of R685 in twisted helix A may tilt from its original position [2], disrupting NCS1 binding [3] (grey dashes)

**Fig. 3:**
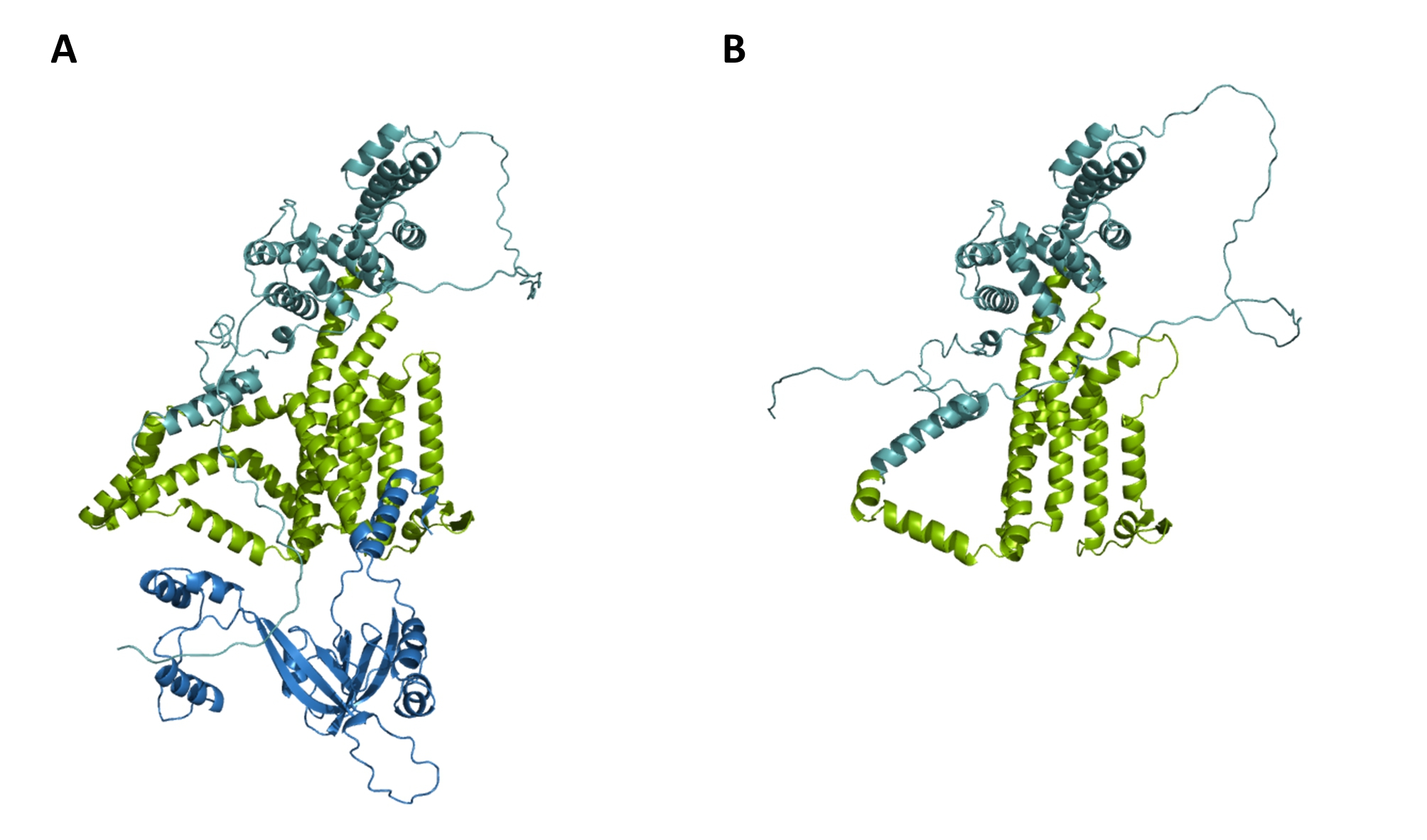
3D modeling and structural analysis of WFS1 p.Phe515LeufsTer28. WFS1 3D model generated from Colabfold. **(A)** WFS1 Wild type **(B)** WFS1 p.Phe515LeufsTer28. Cytoplasmic domain (cyan), TM domain (green), ER-luminal domain (blue). More than one-third of the length of the protein is truncated, including TM domain 7–9 and the ER-luminal domain. Conformational changes of WFS1 mutant (p.Phe515LeufsTer28) were observed

The pathogenicity of p.Arg685Pro as a causative variant for *WFS1*-associated DFNA6/14/18 has previously been reported by several other groups [41, 42]. To improve the structural understanding of p.Arg685Pro and adjacent p.Aarg684Val driven pathogenicity, we first focused their secondary structure. Interestingly, not only the Alphafold server, but the secondary structure simulation servers such as JPRed2 also predicts an alpha-helical structure for Ala684 and Arg685 containing Met683-His692 polypeptide (Fig. 2A) (Additional file 2: Figure S1A). Moreover, this helix (thereafter called helix A) highly interacts with NCS1, well known intra-ER signaling partner of wolframin [43]. Structural prediction of wolframin-NCS1(4GUK) complex using pyDockWEB showed that Arg685 in helix A directly interacts with NCS1 Phe50 based on cation-π interaction (Fig. 2B). Accordingly, p.Arg685Pro substitution directly causes the loss of cation-π interaction between wolframin and NCS1, accompanied with proline mediated helix A destabilization (Fig. 2C). However, p.Ala684Val indirectly interfere wolframin-NCS1 interaction. Non-polar, hydrophobic substitution of Ala684 induces helix destabilization and twists helix A. The side chain of Arg685 in twisted helix A may tilt from its original position, losing the NCS1 binding (Fig. 2D). Accordingly, p.Ala684Val destabilizes the alpha helix and contributes to the loss of WFS1-NCS1 interaction. This is consistent with the prediction servers of regional protein stability, including DynaMut and DynaMut2, demonstrating a negative effect of p.Ala684Val on protein stability (Additional file 3: Table S2). The frameshift variant (p.Phe515LeufsTer28) truncates both transmembrane domains 7–9 and the ER luminal domain (Fig. 3A), severely compromising protein structure stability (Fig. 3B).

### Genotype-phenotype correlation: a systematic review and the present study

Based on a systematic review and the present study, a total of 62 *WFS1* variants causing DFNA6/14/38 or wolfram-like syndrome were identified (Table 3): 7 were in the N-terminal cytoplasmic domain, 12 were in the transmembrane domain, and 43 were in the C-terminal ER luminal domain. The types of variants included missense, inframe deletion, and frameshift in 88.7% (N = 55), 9.7% (N = 6), and 1.6% (N = 1) of cases, respectively. Surprisingly, p.Phe515LeufsTer28 was first identified as a truncated variant related to DFNA6/14/38. The average age of onset for hearing loss in probands with DFNA6/14/38 or wolfram-like syndrome was 15 years (range 1–60). The phenotype of hearing loss was heterogenous; audiometric configuration was primarily specified to low-frequency SNHL (N = 43, 69.4%,) and severity varied from mild to profound. Specifically, *WFS1* variants were present in 14.5% (N = 9) of studies, including 14 patients with severe-to-profound or profound deafness (i.e., possible CI candidates). A total of 11 CI recipients were identified in a systematic review and the present study. CI significantly enhanced auditory performance in *WFS1*-associated DFNA6/14/38. Importantly, only three pathogenic variants (p.Phe515LeufsTer28, p.Ala684Val, and p.Lys836Asn), accounting for *WFS1*-associated DFNA6/14/38, were clustered in CI recipients, suggesting a narrow molecular etiologic spectrum. Furthermore, the p.Ala684Val variant, a known mutational hotspot mutant allele, was confirmed to show severe-to-profound or profound SNHL in all affected patients, and was therefore a strong candidate variant for CI and a genotype-phenotype correlation. Among the 47 probands that were available for ophthalmologic evaluations, 13 cases (27.7%) turned out to have optic atrophy. The causative variants responsible for optic atrophy were p.His313Tyr, p.His323Arg, p.Ala684Val, p.Asn721Tyr, p.Gly780Ser, p.Asp797Tyr, p.Asp797Val, p.Lys836Asn, p.Glu864Lys, and p.Ser869_His872del. In addition, among 47 probands available for the evaluation of diabetic mellitus, 6 (12.8%) were diagnosed. The causative variants associated with diabetic mellitus included p.His313Tyr, p.Ala684Val, p.Asp797Val, p.Val803Met, p.Glu864Lys, and p.Ser869_His872del. No genotype-phenotype correlations were noted for optic atrophy or diabetic mellitus.

**Table 3:** Summary of Clinical features of previously discovered heterozygous variants in the *WFS1* gene associated with DFNA6/14/38

Reference	Genotypes	Hearing Loss				Other phenotypes				Cochlear Implant			
		Onset (yr)	Severity	Configu-ration		Optic atrophy	Diabetes Mellitus	Neurologic Examination		Age (yr)	Outcome	Inheri-tance	Ethnics
Barrett et al. 2009	c.482G > A p.Arg161Gln	N/A	N/A	LF		N/A	N/A	N/A		N/A	N/A	N/A	N/A
Goncavles et al. 2014 [44]	c.511G > A p.Asp171Asn	< 40	moderate	LF		N/A	N/A	Tinnitus		N/A	N/A	N/A	Portuguese
Mohammadi-asl et al. 2021 [45]	c.559 C > T p.Leu187Phe	12–26	severe to profound	N/A		N/A	N/A	normal		N/A	N/A	Familiar	Iranian
Cryns et al. 2002 [46]	c.577 A > C p.Lys193Gln	early	moderate	LF		N/A	N/A	N/A		N/A	N/A	De novo	European
Sloan-Heggen et al. 2016 [32]	c.799G > A p.Asp267Asn	N/A	N/A	N/A		N/A	N/A	N/A		N/A	N/A	N/A	Iran
Kobayashi et al. 2018 [47]	c.908T > C p.Leu303Pro	N/A	mild to moderate	LF, MF		normal	normal	normal		N/A	N/A	Familiar	Japanese
Kobayashi et al. 2018	c.923 C > G p.Ser308Cys	6–16	moderate	LF, MF		normal	normal	Vertigo dizziness		N/A	N/A	Familiar	Japanese
Majander et al. 2022 [48]	c.937 C > T p.His313Tyr	7	N/A	N/A		+	+	Learning disability		N/A	N/A	De novo	British
Majander et al. 2022	c.968 A > G p.His323Arg	4–30	N/A	N/A		+	normal	normal		N/A	N/A	Familiar	British
Sloan-Heggen et al. 2015	c.1072G > A p.Val358Met	N/A	N/A	N/A		N/A	N/A	N/A		N/A	N/A	N/A	Iran
Choi et al. 2013 [49]	c.1235T > C p.Val412Ala	N/A	N/A	N/A		N/A	N/A	N/A		N/A	N/A	N/A	Korean
Wang et al. 2019 [50]	c.1235T > C p.Val412Ala	N/A	N/A	N/A		N/A	N/A	N/A		N/A	N/A	De novo	Chinese
Smith et al. 2004 [51]	c.1371G > T p.Arg457Ser	N/A	N/A	LF		N/A	N/A	N/A		N/A	N/A	N/A	N/A
In this study	c.1544_1545 insA p.Phe515Leu fsTer28	4	profound	HF		normal	normal	normal		56	Improved word and sentence identification	De novo	Korean
Smith et al. 2004	c.1554G > A p.Met518Ile	N/A	N/A	LF		N/A	N/A	N/A		N/A	N/A	N/A	N/A
Smith et al. 2004	c.1669 C > T p.Leu557Phe	N/A	N/A	LF		N/A	N/A	N/A		N/A	N/A	N/A	N/A
Smith et al. 2004	c.1805 C > T p.Ala602Val	N/A	N/A	LF		N/A	N/A	N/A		N/A	N/A	N/A	N/A
Kobayashi et al. 2018	c.1846G > T p.Ala616Ser	19	moderate	HF		normal	normal	Vertigo dizziness		N/A	N/A	Familiar	Japanese
Smith et al. 2004	c.1871T > C p.Val624Ala	N/A	N/A	LF		N/A	N/A	N/A		N/A	N/A	N/A	N/A
Komatsu et al. 2002 [52]	c.1901 A > C p.Lys634Thr	N/A	moderate	LF		normal	normal	normal		N/A	N/A	Familiar	Japanese
Wei et al. 2014 [53]	c.1957 C > T p.Arg653Cys	N/A	mild to severe	LF		normal	normal	normal		N/A	N/A	Familiar	Chinese
Kobayashi et al. 2018	c.1982 A > G p.Asn661Ser	6	moderate	LF		normal	normal	normal		N/A	N/A	Familiar	Japanese
Tsai et al. 2007 [54]	c.2005T > C p.Tyr669His	< 22	moderate	LF		normal	normal	normal		N/A	N/A	Familiar	Taiwanese
Liu et al. 2005 [55]	c.2016G > T p.Leu672=	N/A	N/A	LF		N/A	N/A	N/A		N/A	N/A	N/A	Chinese
Li et al. 2021 [56]	c.2020G > T p.Gly674Trp	19–30	mild to profound	LF, Flat		normal	normal	normal		N/A	N/A	Familiar	Chinese
Cryns et al. 2002	c.2021G > A p.Gly674Glu	early	moderate to severe	LF		N/A	N/A	N/A		N/A	N/A	Familiar	Netherland
Cryns et al. 2002	c.2021G > T p.Gly674Val	early	moderate to severe	LF		N/A	N/A	N/A		N/A	N/A	Familiar	Netherland
Kobayashi et al. 2018	c.2027G > A p.Arg676His	6	severe	flat		normal	normal	normal		N/A	N/A	familiar	Japanese
Wei et al. 2014	c.2036_2038 delAGG p.Glu689del	N/A	mild to severe	LF		normal	normal	normal		N/A	N/A	Familiar	Chinese
Kobayashi et al. 2018	c.2045 A > G p.Asn682Ser	4	moderate	LF		normal	normal	normal		N/A	N/A	De novo	Japanese
Rendtorff et al. 2011 [19]	c.2051 C > T p.Ala684Val	early	severe to profound	Flat, LF, MF		+	normal	N/A		57	Considerably improved hearing	De novo Familiar	Caucasian
Kobayashi et al. 2018	c.2051 C > T p.Ala684Val	< 1	Profound	flat		normal	normal	normal		N/A	N/A	De novo	Japanese
Guan et al. 2020 [57]	c.2051 C > T p.Ala684Val	2–9	Profound	Flat		normal	normal	normal		< 3	Language ability improved	De novo	Chinese
Majander et al. 2022	c.2051 C > T p.Ala684Val	4–60	N/A	N/A		+/-	+/-	normal		N/A	N/A	Familiar	British
Lin et al. 2022 [58]	c.2051 C > T p.Ala684Val	< 3	Profound	flat		N/A	N/A	normal		< 3	SRT 30dB, WRS92% at 35dBHL, CAP/SIR score 6/5	De novo	Taiwanese
In this study	c.2051 C > T p.Ala684Val	< 1	Severe	flat		normal	normal	normal		29mo	CAP score 3 It-MAIS 38/40	De novo	Korean
In this study	c.2051 C > T p.Ala684Val	< 1	Severe	flat		normal	normal	normal		Scheduled	N/A	De novo	Korean
Bramhall et al. 2008 [41]	c.2053G > C p.Arg685Pro	< 4	moderate to severe	LF		N/A	N/A	normal		N/A	N/A	Familiar	Caucasian
Sun et al. 2011 [24]	c.2086 C > T p.His696Tyr	5–28	mild to profound	LF, flat		normal	normal	Vertigo dizziniss		N/A	N/A	Familiar	Chinese
Bespalova et al. 2001 [28]	c.2096 C > T p.Thr699Met	< 25	moderate	LF		normal	normal	normal		N/A	N/A	N/A	Netherland
Sun et al. 2011	c.2108G > A p.Arg703His	7–50	N/A	LF		normal	normal	normal		N/A	N/A	De novo	Chinese
Kunz et al. 2003 [59]	c.2115G > C p.Lys705Asn	< 1	moderate	LF		N/A	N/A	N/A		N/A	N/A	Familiar	Germans
Sloan-Heggen et al. 2016	c.2137_2139 delGAC p.Asp713del	N/A	N/A	N/A		N/A	N/A	N/A		N/A	N/A	N/A	Iranian
Sloan-Heggen et al. 2016	c.2141 A > T p.Asn714Ile	N/A	N/A	N/A		N/A	N/A	N/A		N/A	N/A	N/A	Iranian
Sivakumaran et al. 2004 [60]	c.2146G > A p.Ala716Thr	N/A	N/A	LF		N/A	N/A	N/A		N/A	N/A	N/A	N/A
Bespalova et al. 2011	c.2146G > A p.Ala716Thr	< 10	moderate to severe	LF		normal	normal	normal		N/A	N/A	Familiar	Netherland Irish
Kobayashi et al. 2018	c.2146G > A p.Ala716Thr	< 15	moderate	LF		normal	normal	normal		N/A	N/A	familiar	Japanese
Majander et al. 2022	c.2161 A > T p.Asn721Tyr	50	N/A	N/A		+	normal	nystagmus		N/A	N/A	Familiar	British
Kobayashi et al. 2018	c.2185G > A p.Asp729Asn	< 28	moderate	HF		normal	normal	Vertigo dizziness		N/A	N/A	Familiar De novo	Japanese
Liu et al. 2005	c.2209G > A p.Glu737Lys	N/A	N/A	LF		N/A	N/A	N/A		N/A	N/A	N/A	Chinese
Sloan-Heggen et al. 2015	c.2282 C > T p.Ala761Val	N/A	N/A	N/A		N/A	N/A	N/A		N/A	N/A	N/A	Iranian
Cryns et al. 2002	c.2300–2302 del p.Iledel767	early	N/A	LF		N/A	N/A	N/A		N/A	N/A	N/A	Netherland
Gurtler et al. 2005 [61]	c.2311G > C p.Asp771His	5–20	moderate to profound	LF		N/A	N/A	N/A		N/A	N/A	Familiar	Swiss
Bespalova et al. 2011	c.2335G > A p.Val779Met	N/A	N/A	LF		normal	normal	normal		N/A	N/A	De novo	Americans
Rendtorff et al. 2011	c.2338G > C p.Gly780Ser	congenital	profound	N/A		+	normal	N/A		N/A	N/A	Familiar	Caucasian
Kobayashi et al. 2018	c.2385G > C p.Glu795Asp	6	moderate	LF, flat		normal	normal	normal		N/A	N/A	De novo	Japanese
Bai et al. 2014 [62]	c.2389G > A p.Asp797Asn	1–17	severe to profound	HF, flat		normal	normal	normal		N/A	N/A	familiar	Chinese
Cheng et al. 2018 [63]	c.2389G > A p.Asp797Asn	1–65	mild to profound	flat		normal	normal	normal		N/A	N/A	familiar	Chinese
Rendtorff et al. 2011	c.2389G > T p.Asp797Tyr	3–4	severe to profound	flat		+	normal	normal		N/A	N/A	Familiar	Caucasian
Majander et al. 2022	c.2390 A > T p.Asp797Val	45	N/A	N/A		+	+	normal		N/A	N/A	Familiar	British
Deng et al. 2020 [64]	c.2407G > A p.Val803Met	32–44	mild to severe	HF		normal	+	Demyelinating disorders		N/A	N/A	Familiar	Chinese
Cryns et al. 2003 [65]	c.2419 A > C p.Ser807Arg	early	N/A	LF		N/A	N/A	N/A		N/A	N/A	Familiar	British
Bespalova et al. 2011	c.2486T > C p.Leu829Pro	6–32	moderate	LF		normal	normal	normal		N/A	N/A	N/A	Americans
Cryns et al. 2003	c.2492G > A p.Gly831Asp	< 20	moderate	LF		N/A	N/A	normal		N/A	N/A	N/A	Americans
Fujikawa et al. 2010 [66]	c.2507 A > C p.Lys836Thr	2–10	moderate	LF, MF		normal	normal	normal		N/A	N/A	Familiar	Japanese
Kobayashi et al. 2018	c.2507 A > C p.Lys836Thr	6–28	mild to severe	LF, MF		normal	normal	Vertigo dizziness		N/A	N/A	familiar	Japanese
Hogewind et al. 2010 [67]	c.2508G > C p.Lys836Asn	8–14	severe	flat		+	normal	normal		N/A	83% speech recognition at 70dB SPL	Familiar	Netherland
Kobayashi et al. 2018	c.2508G > C p.Lys836Asn	5–30	moderate to profound	LF, flat		normal	normal	normal		N/A	N/A	familiar	Japanese
Mair et al. 2022 [68]	c.2508G > T p.Lys836Asn	28	moderate to severe	LF		+	normal	normal		N/A	N/A	familiar	Greek
Noguchi et al. 2005 [69]	c.2530G > A p.Ala844Thr	< 6	moderate	LF		normal	normal	normal		N/A	N/A	familiar	Japanese
Gurtler et al. 2005	c.2576G > C p.Arg859Pro	5–30	moderate	LF		N/A	N/A	N/A		N/A	N/A	Familiar	American
Hildebrand et al. 2008 [70]	c.2576G > C p.Arg859Gln	2–45	mild to moderate	LF		N/A	N/A	Parkinson disease		-	-	Familiar	American
Eiberg et al. 2006 [23]	c.2590G > A p.Glu864Lys	4	moderate to severe	LF, flat		+	+/-	normal		N/A	N/A	Familiar	Denmark
Fukuoka et al. 2007 [71]	c.2590G > A p.Glu864Lys	4	moderate to severe	LF		normal	normal	normal		N/A	N/A	familiar	Japanese
Kobayashi et al. 2018	2590G > A p.Glu864Lys	3–7	moderate to profound	LF, MF, flat		+	normal	normal		N/A	N/A	familiar	Japanese
Guan et al. 2020	c.2590G > A p.Glu864Lys	7	mild to moderate	LF		normal	normal	normal		N/A	N/A	De novo	Chinese
Liu et al. 2005	c.2596G > A p.Asp866Asn	N/A	N/A	LF		N/A	N/A	N/A		N/A	N/A	N/A	Chinese
Sloan-Heggen et al. 2016	c.2603G > A p.Arg868His	N/A	N/A	N/A		N/A	N/A	N/A		N/A	N/A	N/A	Iranian
Abu-El-Haija et al. 2021 [27]	c.2605_2616 del p.Ser869_ His872del	3–4	N/A	LF		+	+	normal		N/A	N/A	Familiar	Irish

LF: low-frequency sensorineural hearing loss, MF: middle-frequency sensorineural hearing loss, HF: high-frequency sensorineural hearing loss, N/A: not available;

## Discussion

We expanded the genotypic spectrum of *WFS1* heterozygous variants underlying DFNA6/14/38. Three-dimensional modeling and structural analysis revealed the pathogenicity of mutant WFS1, providing a theoretical basis for WFS1-NCS1 interactions. Based on a systematic review, we presented a range of phenotypic traits for *WFS1* heterozygous variants and demonstrated favorable functional CI outcomes. Remarkably, p.Ala684Val in WFS1 is associated with early-onset severe-to-profound SNHL, rendering it a strong potential marker for CI candidates.

The impact of deafness-causing variants on protein structure has been investigated using structural modeling, which can be used to predict pathogenicity [72]. Ala684 is located within helix A, and alanine is the most common amino acid in helix-formation. In contrast, valine is unfavorable for alpha-helical structure due to its hydrophobic side chain. Several approaches have shown that valine has a helix-destabilizing effect, whereas alanine is a strong helix former [73, 74]. Indeed, valine is often found in β-strands and in transmembrane alpha-helices that interact specifically with lipid chains, and is rarely found in alpha-helices elsewhere. In WFS1 protein, valine is found primarily in transmembrane alpha-helices that interact with the bilipid layer of membrane and β-strands, whereas intra-luminal alpha helices do not have any valine (Additional file 2: Figure S1B). Thus, the non-polar, hydrophobic substitution of Ala684 (p.Ala684Val) may induce helix destabilization and distort helix A. To further elucidate how instability of helix A leads to pathogenicity, a WFS1-NCS1 interaction model was generated. Because helix A may be responsible for intra-ER signaling with respect to NCS1 interactions, p.Aal684Val in WFS1 may lose regular WFS1-NCS1 interactions, even with undamaged ER membrane trafficking. Supporting this, p.Arg685Pro is adjacent to p.Aal684Val and not only disrupts WFS1-NCS1 interaction but also reduces the stability of helix A itself. The proline in helices, a well-known helix terminator, usually kinks or breaks a helix [75]. Therefore, p.Arg685Pro is expected to disrupt helix A and affect its integrity for C-terminal signal transduction. This can be confirmed through biochemical assays to characterize WFS1-NCS1 interaction. Interestingly, p.Phe515LeufsTer28 was first identified as a truncated variant related to DFNA6/14/38. It is clear that p.Phe515LeufsTer28 truncates transmembrane domain 7–9 and the ER-luminal domain, possibly impairing membrane localization and C-terminal signal transduction. The premature stop codon of p.Phe515LeufsTer28 is located before the penultimate exon, which is predicted to undergo NMD in vivo. The autosomal dominant p.Phe515LeufsTer28 variant was hypomorph, and the dominant phenotype is likely to be due to haploinsufficiency, which may be confirmed by further research.

Numerous studies have carried out structural analysis of ADNSHL-associated *WFS1* variants residing in the ER luminal domain [17, 56, 76, 77]. Variants in p.Gly674 (p.Gly674Trp, p.Gly674Glu, p.Gly674Val, and p.Gly674Arg) break the hydrogen bonds between p.Gly674 and p.Thr663, compromising structural stability [56]. Likewise, p.Gly736Asp alters hydrogen bonds and is associated with degeneration of the helix structure (77). p.Glu809Lys and p.Glu830Ala alter polarity and hydrophobicity, with a considerable impact on the surface properties and solvent accessibility of wolframin [76]. Importantly, several functional studies have demonstrated the effect of structural instability caused by variants in the ER luminal domain. Wolframin is likely to undergo misfolding due to *WFS1* variants in the ER-luminal domain, shortening its half-life and causing rapid degradation [19]. Indeed, the missense variant in the ER-luminal domain (p.Ala684Val) is known to cause misfolding of wolframin protein, as shown by its reduced expression level due to rapid degradation [19]. Further, p.Gly695Val and p.Pro724Leu hinder membrane translocation because of their aggregation in the ER [78]. ER-localized Na+/K + ATPase beta-1 subunit (ATP1B1) binds to the ER-luminal domain of wolframin [79]. Thus, Na+/K + ATPase deficit due to variants in the ER-luminal domain impairs C-terminal signal transduction, which is essential for ER stress and apoptosis. Increased ER stress, due to mutant wolframin, has shown to cause apoptosis of cochlear cells, resulting in hearing loss [80]. Collectively, these structural and molecular phenomena may increase ER stress and disturb calcium homeostasis in the inner ear, as well as the maintenance of endo-cochlear potential, with a consequent deterioration in hearing.

The results of this study, as well as a systematic review, demonstrated favorable CI outcomes for *WFS1*-associated ADNSHL. A total of 11 CI recipients were included; most significantly improved their language skills after surgery. We also observed that auditory performance significantly improved, even at postoperative 3 months. Loss of spiral ganglion neurons (SGNs) is an important determinant of CI outcome. Taking into account the classic SGNs hypothesis [81], *WFS1*-associated ADNSHL was expected to have good CI outcomes, due to the spatial expression of wolframin in presynaptic regions in the inner ear. Remarkably, the molecular genetic etiology for CI recipients clustered around three pathogenic variants (p.Phe515LeufsTer28, p.Ala684Val, and p.Lys836Asn), indicating a narrow molecular etiological spectrum. Specifically, the p.Ala684Val variant was identified in 81.8% of cases (9 out of 11), indicating that it may be a strong CI marker. Likewise, p.Ala684Val in WFS1 was responsible for early-onset severe-to-profound deafness in all affected patients, suggesting a close genotype-phenotype correlation. p.Ala684Val, a known mutational hotspot allele, often arose from *de novo* variants. The mode of inheritance of p.Ala684Val appears to be consistent among ethnic backgrounds, including Caucasian, Japanese, Chinese, and Taiwanese patients [19, 47, 57, 58]. Putatively, fetuses with developmentally induced *de novo* variants may be at risk for more severe auditory phenotypes, necessitating CI at an early stage.

## Conclusion

We expanded the genotypic spectrum of *WFS1* heterozygous variants underlying DFNA6/14/38, and revealed their pathogenicity upon 3D modeling and structural analysis. Non-polar, hydrophobic substitution of Ala684 (p.Ala684Val) destabilized helix A and contributed to loss of WFS1-NCS1 interaction, which is required for C-terminal signal transduction. The results of this study, along with a systematic review, demonstrated favorable functional outcomes of cochlear implantation in *WFS1*-associated ADNSHL. Remarkably, the molecular genetic etiology for CI recipients clustered around only three pathogenic variants, indicating a narrow molecular etiological spectrum. Specifically, p.Ala684Val in *WFS1* is associated with early-onset severe-to-profound or profound SNHL, indicating that this variant may be a strong CI marker.

## Electronic supplementary material

Below is the link to the electronic supplementary material.

**Additional File 1**: Table S1

**Additional File 1**: Figure S1

**Additional File 1**: Table S2

## Data Availability

The datasets generated and/or analyzed during the current study have been submitted in ClinVar under accession number SCV002818466 and VCV000030556.25 (https://www.ncbi.nlm.nih.gov/clinvar/variation/30556/). All other relevant data of this study are available within the article and its Supplementary Material. Individual-level whole-exome sequence data are not publicly available due to lack of ethical approval but are available from the corresponding author on reasonable request.

## List of abbreviations

WFS1: Wolfram syndrome type 1 gene
ER: Endoplasmic reticulum
TM: Transmembrane
SNHL: Sensorineural hearing loss
ADNSHL: Autosomal dominant nonsyndromic hearing loss
CAP: Categories of Auditory Perception
IT-MAIS: Infant-Toddler Meaningful Auditory Integration Scale
SELSI: Sequenced Language Scale for Infants
K-CID: Korean version of the Central Institute of Deafness
GERP: Genomic Evolutionary Rate Profiling
CADD: Combined Annotation Dependent Depletion
REVEL: Rare Exome Variant Ensemble Learner
LF: low-frequency sensorineural hearing loss
MF: middle-frequency sensorineural hearing loss
HF: high-frequency sensorineural hearing loss
CI: Cochlea implantation

## References

[1] Davis A, Davis K. Descriptive epidemiology of childhood hearing impairment.Comprehensive handbook of pediatric audiology. 2011:85–111.

[2] Morton CC, Nance WE (2006). Newborn hearing screening — a Silent Revolution. N Engl J Med.

[3] Van Camp G. Hereditary hearing loss homepage. URL: https://hereditaryhearingloss.org. 2006.

[4] Jo HD, Han JH, Lee SM, Choi DH, Lee S-Y, Choi BY (2022). Genetic load of alternations of transcription factor genes in non-syndromic deafness and the Associated Clinical Phenotypes: experience from two Tertiary Referral Centers. Biomedicines.

[5] Lee S-Y, Choi HB, Park M, Choi IS, An J, Kim A (2021). Novel KCNQ4 variants in different functional domains confer genotype-and mechanism-based therapeutics in patients with nonsyndromic hearing loss. Exp Mol Med.

[6] Lee S-Y, Han JH, Kim BJ, Oh SH, Lee S, Oh D-Y (2019). Identification of a potential founder effect of a novel PDZD7 variant involved in moderate-to-severe sensorineural hearing loss in Koreans. Int J Mol Sci.

[7] Lee S-Y, Joo K, Oh J, Han JH, Park H-R, Lee S (2020). Severe or profound sensorineural hearing loss caused by novel USH2A variants in Korea: potential genotype-phenotype correlation. Clin Exp Otorhinolaryngol.

[8] Lee S-Y, Yoo HS, Han JH, Lee DH, Park SS, Suh MH et al. Novel Molecular Genetic Etiology of Asymmetric Hearing Loss: Autosomal-Dominant LMX1A Variants.Ear and Hearing. 2022:10.1097. 10.1097/AUD.0000000000001237 35711095

[9] Lee SY, Han JH, Carandang M, Kim MY, Kim B, Yi N (2020). Novel genotype–phenotype correlation of functionally characterized LMX1A variants linked to sensorineural hearing loss. Hum Mutat.

[10] Oziębło D, Lee SY, Leja ML, Sarosiak A, Bałdyga N, Skarżyński H (2022). Update on CD164 and LMX1A genes to strengthen their causative role in autosomal dominant hearing loss. Hum Genet.

[11] Lee S-Y, Oh D-Y, Han JH, Kim MY, Kim B, Kim BJ (2020). Flexible real-time polymerase chain reaction-based platforms for detecting deafness mutations in koreans: a proposed guideline for the etiologic diagnosis of auditory neuropathy spectrum disorder. Diagnostics.

[12] Lee S-Y, Shim YJ, Han J-H, Song J-J, Koo J-W, Oh SH (2020). The molecular etiology of deafness and auditory performance in the postlingually deafened cochlear implantees. Sci Rep.

[13] Delmaghani S, El-Amraoui A (2020). Inner ear gene therapies take off: current promises and future challenges. J Clin Med.

[14] Hofmann S, Philbrook C, Gerbitz K-D, Bauer MF (2003). Wolfram syndrome: structural and functional analyses of mutant and wild-type wolframin, the WFS1 gene product. Hum Mol Genet.

[15] Matsunaga K, Tanabe K, Inoue H, Okuya S, Ohta Y, Akiyama M (2014). Wolfram syndrome in the japanese population; molecular analysis of WFS1 gene and characterization of clinical features. PLoS ONE.

[16] Omasits U, Ahrens CH, Müller S, Wollscheid B (2014). Protter: interactive protein feature visualization and integration with experimental proteomic data. Bioinformatics.

[17] Qian X, Qin L, Xing G, Cao X (2015). Phenotype prediction of pathogenic nonsynonymous single nucleotide polymorphisms in WFS1. Sci Rep.

[18] Takeda K, Inoue H, Tanizawa Y, Matsuzaki Y, Oba J, Watanabe Y (2001). WFS1 (Wolfram syndrome 1) gene product: predominant subcellular localization to endoplasmic reticulum in cultured cells and neuronal expression in rat brain. Hum Mol Genet.

[19] Rendtorff ND, Lodahl M, Boulahbel H, Johansen IR, Pandya A, Welch KO (2011). Identification of p. A684V missense mutation in the WFS1 gene as a frequent cause of autosomal dominant optic atrophy and hearing impairment. Am J Med Genet Part A.

[20] Hu K, Zatyka M, Astuti D, Beer N, Dias RP, Kulkarni A (2022). WFS1 protein expression correlates with clinical progression of optic atrophy in patients with Wolfram syndrome. J Med Genet.

[21] Cryns K, Thys S, Van Laer L, Oka Y, Pfister M, Van Nassauw L (2003). The WFS1 gene, responsible for low frequency sensorineural hearing loss and Wolfram syndrome, is expressed in a variety of inner ear cells. Histochem Cell Biol.

[22] Suzuki N, Hosoya M, Oishi N, Okano H, Fujioka M, Ogawa K (2016). Expression pattern of wolframin, the WFS1 (Wolfram syndrome-1 gene) product, in common marmoset (Callithrix jacchus) cochlea. NeuroReport.

[23] Eiberg H, Hansen L, Kjer B, Hansen T, Pedersen O, Bille M (2006). Autosomal dominant optic atrophy associated with hearing impairment and impaired glucose regulation caused by a missense mutation in the WFS1 gene. J Med Genet.

[24] Sun Y, Cheng J, Lu Y, Li J, Lu Y, Jin Z (2011). Identification of two novel missense WFS1 mutations, H696Y and R703H, in patients with non-syndromic low-frequency sensorineural hearing loss. J Genet Genomics.

[25] Inoue H, Tanizawa Y, Wasson J, Behn P, Kalidas K, Bernal-Mizrachi E (1998). A gene encoding a transmembrane protein is mutated in patients with diabetes mellitus and optic atrophy (Wolfram syndrome). Nat Genet.

[26] Rigoli L, Lombardo F, Di Bella C (2011). Wolfram syndrome and WFS1 gene. Clin Genet.

[27] Abu-El‐Haija A, McGowan C, Vanderveen D, Bodamer O, Autosomal‐dominant (2021). WFS1‐related disorder—report of a novel WFS1 variant and review of the phenotypic spectrum of autosomal recessive and dominant forms. Am J Med Genet Part A.

[28] Bespalova IN, Van Camp G, Bom JH, Brown S, Cryns DJ, DeWan K (2001). Mutations in the Wolfram syndrome 1 gene (WFS1) are a common cause of low frequency sensorineural hearing loss. Hum Mol Genet.

[29] Clark JG (1981). Uses and abuses of hearing loss classification. Asha.

[30] Kral A, O’Donoghue GM (2010). Profound deafness in childhood. N Engl J Med.

[31] Lee S-Y, Choi BY (2021). Potential implications of slim modiolar electrodes for severely malformed cochleae: a comparison with the straight array with circumferential electrodes. Clin Exp Otorhinolaryngol.

[32] Sloan-Heggen CM, Bierer AO, Shearer AE, Kolbe DL, Nishimura CJ, Frees KL (2016). Comprehensive genetic testing in the clinical evaluation of 1119 patients with hearing loss. Hum Genet.

[33] Oza AM, DiStefano MT, Hemphill SE, Cushman BJ, Grant AR, Siegert RK (2018). Expert specification of the ACMG/AMP variant interpretation guidelines for genetic hearing loss. Hum Mutat.

[34] Patel MJ, DiStefano MT, Oza AM, Hughes MY, Wilcox EH, Hemphill SE (2021). Disease-specific ACMG/AMP guidelines improve sequence variant interpretation for hearing loss. Genet Sci.

[35] Jumper J, Evans R, Pritzel A, Green T, Figurnov M, Ronneberger O (2021). Highly accurate protein structure prediction with AlphaFold. Nature.

[36] Varadi M, Anyango S, Deshpande M, Nair S, Natassia C, Yordanova G, et al. AlphaFold protein structure database: massively expanding the structural coverage of protein-sequence space with high-accuracy models. Nucleic acids research; 2021. 10.1093/nar/gkab1061 PMC8728224 34791371

[37] Mirdita M, Schütze K, Moriwaki Y, Heo L, Ovchinnikov S, Steinegger M. ColabFold: making protein folding accessible to all.Nature Methods. 2022:1–4. 10.1038/s41592-022-01488-1 PMC9184281 35637307

[38] Jiménez-García B, Pons C, Fernández-Recio J (2013). pyDockWEB: a web server for rigid-body protein–protein docking using electrostatics and desolvation scoring. Bioinformatics.

[39] Waschbisch A, Volbers B, Struffert T, Hoyer J, Schwab S, Bardutzky J (2011). Primary diagnosis of Wolfram syndrome in an adult patient—case report and description of a novel pathogenic mutation. J Neurol Sci.

[40] Xavier J, Bourvis N, Tanet A, Ramos T, Perisse D, Marey I (2016). Bipolar disorder type 1 in a 17-year-old girl with Wolfram syndrome. J Child Adolesc Psychopharmacol.

[41] Bramhall NF, Kallman JC, Verrall AM, Street VA (2008). A novel WFS1 mutation in a family with dominant low frequency sensorineural hearing loss with normal VEMP and EcochG findings. BMC Med Genet.

[42] Duzkale H, Shen J, McLaughlin H, Alfares A, Kelly M, Pugh T (2013). A systematic approach to assessing the clinical significance of genetic variants. Clin Genet.

[43] Angebault C, Fauconnier J, Patergnani S, Rieusset J, Danese A, Affortit CA (2018). ER-mitochondria cross-talk is regulated by the Ca2 + sensor NCS1 and is impaired in Wolfram syndrome. Sci Signal.

[44] Goncalves A, Matos T, Simoes-Teixeira H, Machado MP, Simao M, Dias O (2014). WFS1 and non-syndromic low-frequency sensorineural hearing loss: a novel mutation in a portuguese case. Gene.

[45] Mohammadi Asl J, Saki N, Dehdashtiyan M, Neissi M, Ghanbari Mardasi F (2021). Identification of a Novel WFS1 mutation using the whole exome sequencing in an iranian pedigree with autosomal Dominant hearing loss. Iran J Otorhinolaryngol.

[46] Cryns K, Pfister M, Pennings RJ, Bom SJ, Flothmann K, Caethoven G (2002). Mutations in the WFS1 gene that cause low-frequency sensorineural hearing loss are small non-inactivating mutations. Hum Genet.

[47] Kobayashi M, Miyagawa M, Nishio S-y, Moteki H, Fujikawa T, Ohyama K (2018). WFS1 mutation screening in a large series of japanese hearing loss patients: massively parallel DNA sequencing-based analysis. PLoS ONE.

[48] Majander A, Jurkute N, Burté F, Brock K, João C, Huang H et al. WFS1-Associated Optic Neuropathy: Genotype-Phenotype Correlations and Disease Progression.American Journal of Ophthalmology. 2022. 10.1016/j.ajo.2022.04.003 35469785

[49] Choi BY, Park G, Gim J, Kim AR, Kim B-J, Kim H-S (2013). Diagnostic application of targeted resequencing for familial nonsyndromic hearing loss. PLoS ONE.

[50] Wang Z, Huang C, Sun Y, Lv H, Zhang M, Li X (2019). Novel mutations associated with autosomal–dominant congenital cataract identified in chinese families. Experimental and Therapeutic Medicine.

[51] Smith CJ, Crock PA, King BR, Meldrum CJ, Scott RJ (2004). Phenotype-genotype correlations in a series of wolfram syndrome families. Diabetes Care.

[52] Komatsu K, Nakamura N, Ghadami M, Matsumoto N, Kishino T, Ohta T (2002). Confirmation of genetic homogeneity of nonsyndromic low-frequency sensorineural hearing loss by linkage analysis and a DFNA6/14 mutation in a japanese family. J Hum Genet.

[53] Wei Q, Zhu H, Qian X, Chen Z, Yao J, Lu Y (2014). Targeted genomic capture and massively parallel sequencing to identify novel variants causing chinese hereditary hearing loss. J translational Med.

[54] Tsai H-T, Wang Y-P, Chung S-F, Lin H-C, Ho G-M, Shu M-T (2007). A novel mutation in the WFS1gene identified in a taiwanese family with low-frequency hearing impairment. BMC Med Genet.

[55] 小林正史. WFS1 mutation screening in a large series of japanese hearing loss patients: massively parallel DNA sequencing-based analysis (日本人難聴患者における WFS1 遺伝子変異のスクリーニング). Shinshu University Library; 2019.

[56] Li J, Xu H, Sun J, Tian Y, Liu D, Qin Y et al. Missense Variant of Endoplasmic Reticulum Region of WFS1 Gene Causes Autosomal Dominant Hearing Loss without Syndromic Phenotype. BioMed Research International. 2021;2021. 10.1155/2021/6624744 PMC8260318 34258273

[57] Guan J, Wang H, Lan L, Wu Y, Chen G, Zhao C (2020). Recurrent de novo WFS1 pathogenic variants in chinese sporadic patients with nonsyndromic sensorineural hearing loss. Mol Genet Genom Med.

[58] Lin P-H, Wu H-P, Wu C-M, Chiang Y-T, Hsu JS, Tsai C-Y (2022). Cochlear implantation outcomes in patients with auditory Neuropathy Spectrum disorder of genetic and non-genetic etiologies: a Multicenter Study. Biomedicines.

[59] Kunz J, Marquez-Klaka B, Uebe S, Volz-Peters A, Berger R, Rausch P (2003). Identification of a novel mutation in WFS1 in a family affected by low-frequency hearing impairment. Mutat Research/Fundamental Mol Mech Mutagen.

[60] Sivakumaran TA, Lesperance MM (2002). A PCR-RFLP assay for the A716T mutation in the WFS1 gene, a common cause of low-frequency sensorineural hearing loss. Genet Test.

[61] Gürtler N, Kim Y, Mhatre A, Schlegel C, Mathis A, Daniels R (2005). Two families with nonsyndromic low-frequency hearing loss harbor novel mutations in Wolfram syndrome gene 1. J Mol Med.

[62] Bai X, Lv H, Zhang F, Liu J, Fan Z, Xu L (2014). Identification of a novel missense mutation in the WFS1 gene as a cause of autosomal dominant nonsyndromic sensorineural hearing loss in all-frequencies. Am J Med Genet Part A.

[63] Cheng H, Zhang Q, Wang W, Meng Q, Wang F, Liu M (2018). Whole exome sequencing identifies a pathogenic mutation in WFS1 in two large chinese families with autosomal dominant all-frequency hearing loss and prenatal counseling. Int J Pediatr Otorhinolaryngol.

[64] Deng H, Zhang J, Zhu F, Deng X, Yuan L (2020). Identification of the rare variant p. Val803Met of WFS1 gene as a cause of Wolfram-like syndrome in a chinese family. Acta Diabetol.

[65] Cryns K, Sivakumaran TA, Van den Ouweland JM, Pennings RJ, Cremers CW, Flothmann K (2003). Mutational spectrum of the WFS1 gene in Wolfram syndrome, nonsyndromic hearing impairment, diabetes mellitus, and psychiatric disease. Hum Mutat.

[66] Fujikawa T, Noguchi Y, Ito T, Takahashi M, Kitamura K (2010). Additional heterozygous 2507A > C mutation of WFS1 in progressive hearing loss at lower frequencies. Laryngoscope.

[67] Hogewind BF, Pennings RJ, Hol FA, Kunst HP, Hoefsloot EH, Cruysberg JR (2010). Autosomal dominant optic neuropathy and sensorineual hearing loss associated with a novel mutation of WFS1. Mol Vis.

[68] Mair H, Fowler N, Papatzanaki ME, Sudhakar P, Maldonado RS. Novel missense WFS1 variant causing autosomal dominant atypical Wolfram syndrome.Ophthalmic Genetics. 2022:1–6. 10.1080/13816810.2022.2068038 35450504

[69] Noguchi Y, Yashima T, Hatanaka A, Uzawa M, Yasunami M, Kimura A (2005). A mutation in Wolfram syndrome type 1 gene in a japanese family with autosomal dominant low-frequency sensorineural hearing loss. Acta Otolaryngol.

[70] Hildebrand MS, Sorensen JL, Jensen M, Kimberling WJ, Smith RJ (2008). Autoimmune disease in a DFNA6/14/38 family carrying a novel missense mutation in WFS1. Am J Med Genet Part A.

[71] Fukuoka H, Kanda Y, Ohta S, Usami S (2007). -i. mutations in the WFS1 gene are a frequent cause of autosomal dominant nonsyndromic low-frequency hearing loss in japanese. J Hum Genet.

[72] Buonfiglio PI, Bruque CD, Lotersztein V, Luce L, Giliberto F, Menazzi S (2022). Predicting pathogenicity for novel hearing loss mutations based on genetic and protein structure approaches. Sci Rep.

[73] Lyu PC, Sherman JC, Chen A, Kallenbach NR. Alpha-helix stabilization by natural and unnatural amino acids with alkyl side chains. Proceedings of the National Academy of Sciences. 1991;88(12):5317-20. 10.1073/pnas.88.12.5317 PMC51863 2052608

[74] Okamoto Y (1994). Helix-forming tendencies of nonpolar amino acids predicted by Monte Carlo simulated annealing. Proteins Struct Funct Bioinform.

[75] Nilsson I, SaÈaÈf A, Whitley P, Gafvelin G, Waller C, von Heijne G (1998). Proline-induced disruption of a transmembrane α-helix in its natural environment. J Mol Biol.

[76] De Franco E, Flanagan SE, Yagi T, Abreu D, Mahadevan J, Johnson MB (2017). Dominant Er stress–inducing Wfs1 mutations underlie a genetic syndrome of neonatal/infancy-onset diabetes, congenital sensorineural deafness, and congenital cataracts. Diabetes.

[77] Torkamandi S, Rezaei S, Mirfakhraie R, Bayat S, Piltan S, Gholami M (2020). A homozygous missense mutation of WFS1 gene causes Wolfram’s syndrome without hearing loss in an iranian family (a report of clinical heterogeneity). J Clin Lab Anal.

[78] Fonseca SG, Fukuma M, Lipson KL, Nguyen LX, Allen JR, Oka Y (2005). WFS1 is a novel component of the unfolded protein response and maintains homeostasis of the endoplasmic reticulum in pancreatic β-cells. J Biol Chem.

[79] Zatyka M, Ricketts C, da Silva Xavier G, Minton J, Fenton S, Hofmann-Thiel S (2008). Sodium-potassium ATPase β1 subunit is a molecular partner of Wolframin, an endoplasmic reticulum protein involved in ER stress. Hum Mol Genet.

[80] Rigoli L, Bramanti P, Di Bella C, De Luca F (2018). Genetic and clinical aspects of Wolfram syndrome 1, a severe neurodegenerative disease. Pediatr Res.

[81] Eppsteiner RW, Shearer AE, Hildebrand MS, DeLuca AP, Ji H, Dunn CC (2012). Prediction of cochlear implant performance by genetic mutation: the spiral ganglion hypothesis. Hear Res.

