# Supplementary material for: WFS1 autosomal dominant variants linked with hearing loss: update on structural analysis and cochlear implant outcome
Source: BMC Med Genomics. 2023 Apr 11;16:79. doi: 10.1186/s12920-023-01506-x (PMC10088283; doi:10.1186/s12920-023-01506-x)
Supplement: Supplementary file 1 — Additional File 1: Table S1 [file 12920_2023_1506_MOESM1_ESM.docx]

**Table S1.** Cochlear implantation outcomes in the present study.

| SH 486 | CAP | Adult Screening Test | | | |  | Speech Perception Test | | |
| --- | --- | --- | --- | --- | --- | --- | --- | --- | --- |
|  |  | Word length  identification | Sentence length  identification | Discrimination of  Second formant | Common sentence  recognition |  | Monosyllabic  words | Bisyllabic  words | Sentences |
| Pre-op | 4 | 80% | 90% | 50% | 37% |  | 28% | 32% | 27% |
| Post-op (3mo) | N/A | 100% | 90% | 90% | 46% |  | 60% | 60% | 60% |

| SH 550 | CAP | It-MAIS | Little-Ears | SELSI | |
| --- | --- | --- | --- | --- | --- |
|  |  |  |  | Receptive language ability | Expressive language ability |
| Pre-op | 1 | 2/40 | 0/35 | 4 (2mo) | 8 (5mo) |
| Post-op  (3mo) | 3 | 38/40 | 14/35 | 13 (7mo) | 14 (8mo) |

CAP: Category of Auditory Performance, It-MAIS: Infant-Toddler Meaningful Auditory Integration Scale, SELSI: Sequenced Language Scale for Infant
