# Supplementary figures and images for: WFS1 autosomal dominant variants linked with hearing loss: update on structural analysis and cochlear implant outcome
Source: BMC Med Genomics. 2023 Apr 11;16:79. doi: 10.1186/s12920-023-01506-x (PMC10088283; doi:10.1186/s12920-023-01506-x)

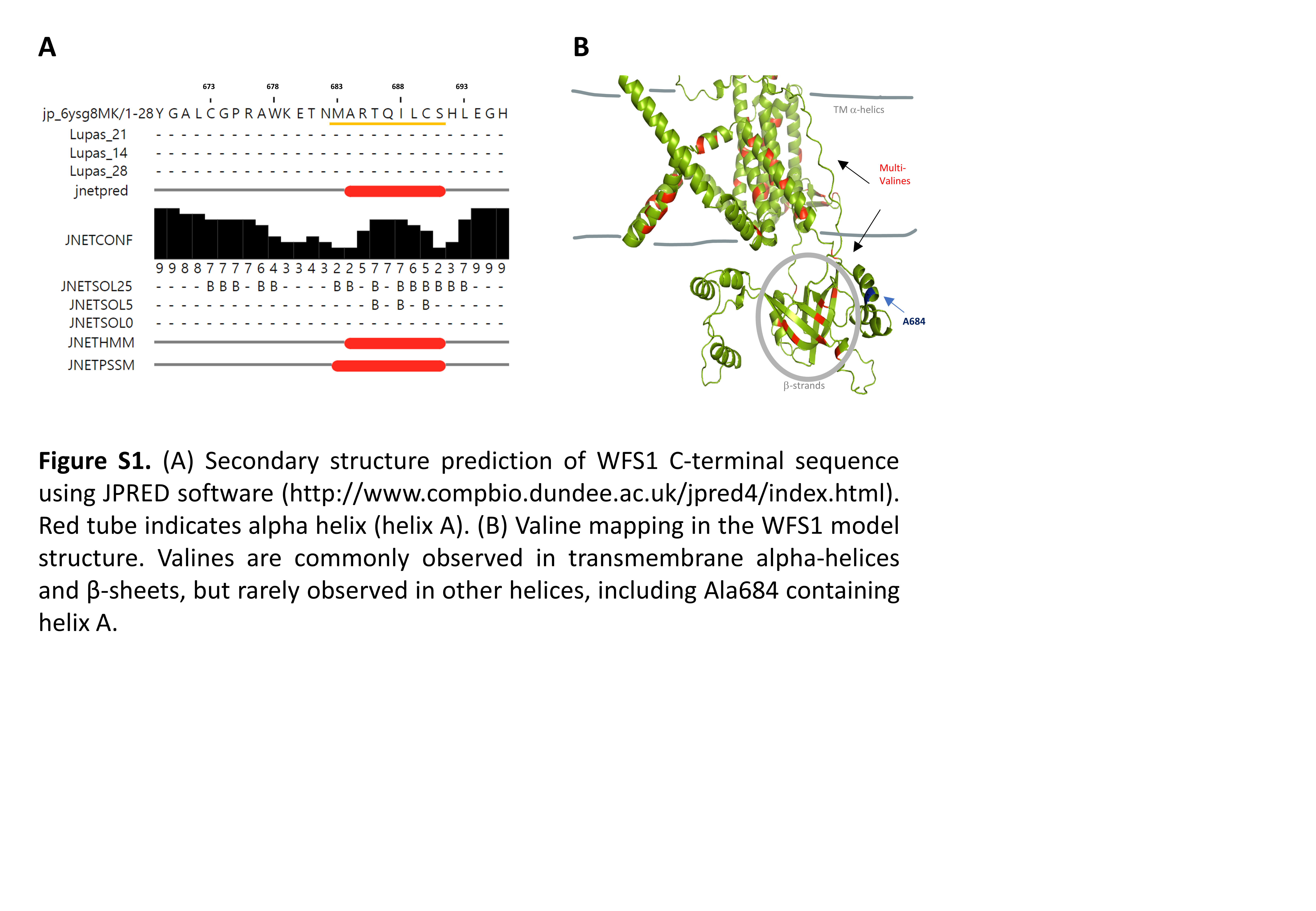

Supplement: Supplementary file 2 — Additional File 1: Figure S1 [file 12920_2023_1506_MOESM2_ESM.tif]
