# Supplementary material for: WFS1 autosomal dominant variants linked with hearing loss: update on structural analysis and cochlear implant outcome
Source: BMC Med Genomics. 2023 Apr 11;16:79. doi: 10.1186/s12920-023-01506-x (PMC10088283; doi:10.1186/s12920-023-01506-x)
Supplement: Supplementary file 3 — Additional File 1: Table S2 [file 12920_2023_1506_MOESM3_ESM.docx]

**Table S2**. The prediction of protein stability and pathogenicity in DynaMut, and Dy-naMut2;

| WFS1 variant | DynaMut $\Delta\Delta$G (kcal/moL) | DynaMut2 $\Delta\Delta$G (kcal/moL) |
| --- | --- | --- |
| p.Ala684Val | -0.270 (Destabilizing) | -0.90 (Destabilizing) |

The difference in Gibbs free energy of folding ($\Delta\Delta G= \Delta G_{WT}-\Delta G_{MT}$)

DynaMut(<http://biosig.unimelb.edu.au/dynamut/>); DynaMut2(http://biosig.unimelb.edu.au/dynamut2).
